# Suppression of human T cell activation by derivatives of glycerol monolaurate

**DOI:** 10.1038/s41598-021-88584-y

**Published:** 2021-04-26

**Authors:** Micaela G. Fosdick, Pratik Rajesh Chheda, Phuong M. Tran, Alex Wolff, Ronal Peralta, Michael Y. Zhang, Robert Kerns, Jon C. D. Houtman

**Affiliations:** 1 Biomedical Sciences Graduate Program, Subprogram in Molecular Medicine, Carver College of Medicine, University of Iowa, 2110 MERF, Iowa City, IA 52242 USA; 2 Department of Microbiology and Immunology, Carver College of Medicine, University of Iowa, Iowa City, USA; 3 Department of Pharmaceutical Sciences and Experimental Therapeutics, College of Pharmacy, University of Iowa, Iowa City, USA

**Keywords:** Lymphocyte activation, Signal transduction

## Abstract

Glycerol monolaurate (GML), a naturally occurring monoglyceride, is widely used commercially for its antimicrobial properties. Interestingly, several studies have shown that GML not only has antimicrobial properties but is also an anti-inflammatory agent. GML inhibits peripheral blood mononuclear cell proliferation and inhibits T cell receptor (TCR)-induced signaling events. In this study, we perform an extensive structure activity relationship analysis to investigate the structural components of GML necessary for its suppression of human T cell activation. Human T cells were treated with analogs of GML, differing in acyl chain length, head group, linkage of acyl chain, and number of laurate groups. Treated cells were then tested for changes in membrane dynamics, LAT clustering, calcium signaling, and cytokine production. We found that an acyl chain with 12–14 carbons, a polar head group, an ester linkage, and a single laurate group at any position are all necessary for GML to inhibit protein clustering, calcium signaling, and cytokine production. Removing the glycerol head group or replacing the ester linkage with a nitrogen prevented derivative-mediated inhibition of protein cluster formation and calcium signaling, while still inhibiting TCR-induced cytokine production. These findings expand our current understanding of the mechanisms of action of GML and the of GML needed to function as a novel immunosuppressant.

## Introduction

Glycerol monolaurate (GML) is a naturally occurring monoglyceride with a 12-carbon acyl chain attached to a glycerol head group via an ester linkage. This compound can be found in concentrations as high as 3 mg/mL in human breast milk^1^ and lauric acid, an analog of GML, makes up ~ 50% of the fatty acids found in triglycerides in coconut oil^2^. GML has broad spectrum antimicrobial properties, microbial referring to fungi, enveloped viruses, gram-positive bacteria, and select gram-negative bacteria^3^. Intriguingly, no resistance has been identified in any susceptible microbe to date. As it is found on the FDA’s Generally Recognized as Safe list, GML has been used commercially as a preservative and emulsifier in products ranging from food items to personal hygiene products. Furthermore, GML has been tested in several clinical trials and in vivo studies for potential clinical applications. Previous studies found that tampons containing GML reduced *Staphylococcus aureus* excreted cytotoxins and pro-inflammatory cytokines associated with toxic shock syndrome^4,5^. In addition, studies in Rhesus monkeys vaginally infected with SIV demonstrated GML prevents mucosal SIV transmission^6,7^. The testing of GML in various treatments raised the question of how this compound interacts with eukaryotic cells needed for pathogen clearance.

In addition to its antimicrobial properties, several studies have demonstrated that in GML can also act as an anti-inflammatory agent. GML inhibits proliferation of peripheral blood mononuclear cells (PBMCs) stimulated with phorbol myristate acetate (PMA) and Toxic Shock Syndrome Toxin 1 (TSST-1)^8^. Further work from our laboratory found that in serum free conditions GML inhibits T cell receptor (TCR)-mediated activation by disrupting lipid membrane dynamics and altering select early signaling events^9,10^. Transmission of signaling pathways downstream of TCR activation relies on the formation of large clusters of critical signaling proteins, such as phosphorylated linker of activated T cells (pLAT). The clustering of LAT is initiated and maintained by the binding of dimerized GRB2 but also appears to be stabilized by alterations in lipid membrane dynamics^11–13^. The clustering of pLAT results in downstream calcium signaling, cytoskeleton rearrangement, and cytokine production; these effects are inhibited by human serum albumin^11,13–15^. GML treatment significantly increases both ordered and disordered lipid membrane domains and significantly decreases pLAT cluster formation. Furthermore, GML suppresses TCR-induced calcium signaling and inhibits cytokine production in a dose dependent manner^10^. GML treatment also disrupts LAT dependent SLP-76 micro clusters which results in altered localization of the ARPC3 subunit and activation of ARP2/3 complex^16^. These changes result in filopodia formation instead of lamellipodia upon TCR activation, and an altered actin arrangement^16^. These studies suggest that GML has an interesting potential therapeutic role as an immune modulating monoglyceride.

In this study, we investigated the structural components required for the ability of GML to impact T cell biology and identify additional analogs of GML with immune-modulating activity. To investigate these questions, we treated human primary T cells with structural analogs of GML with variable chain length, linkage, head group, position, and number of laurate groups. We found that chain length, polarity of head group, ester linkage, and a single laurate group at any position are all necessary for GML to inhibit T cell activation. Together, these studies identify GML and its analogs as a novel class of immune modulating molecules.

## Results

### GML derivatives and T cell viability

**Figure 1:**
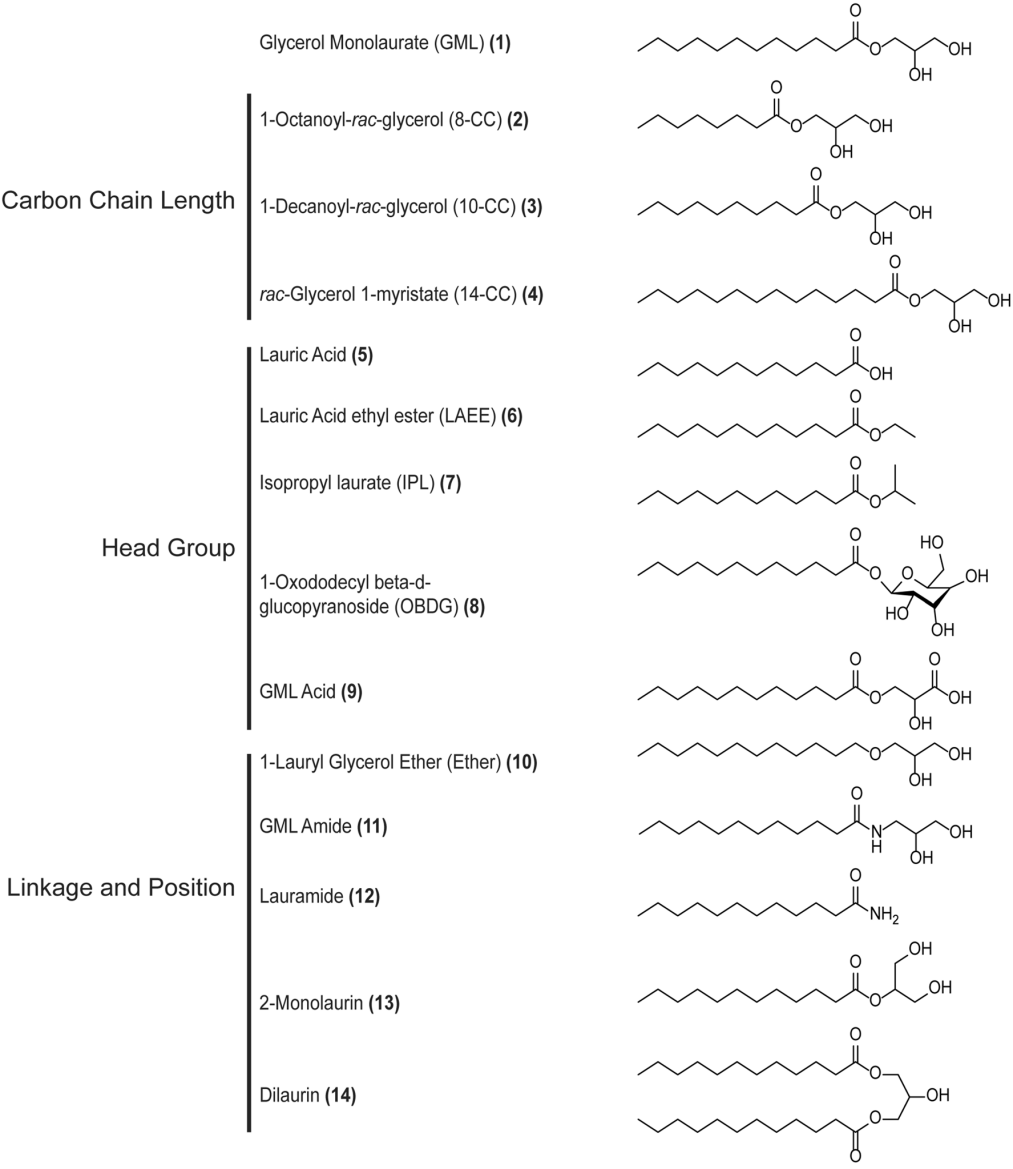
GML analogs used in this study. Structures of compounds used in this study. Analogs varied in chain length, head group, linkage, and position.

**Figure 2:**
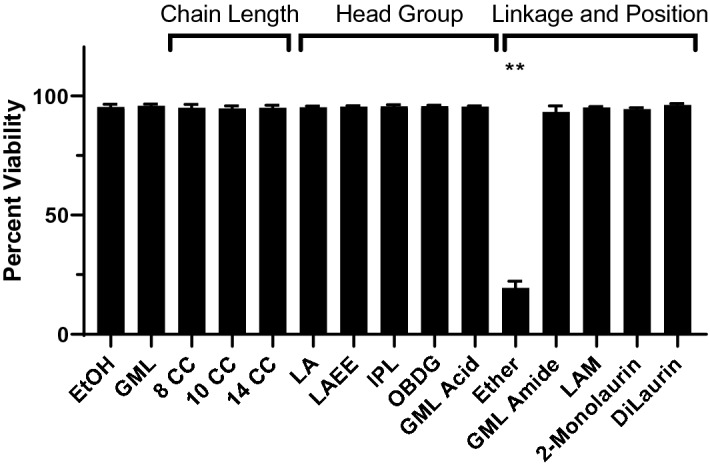
Cell viability assay. Rested Human APBTs were treated with 10ug/mL of designated compound overnight and tested for viability via Trypan Blue exclusion. Data presented is an average percentage of viable cells calculated using (Trypan Blue Positive Cells)/(Trypan Blue Pos. + Trypan Blue Neg.) compiled from 3 independent experiments. Statistical significance was determined using a Repeated Measures One-Way ANOVA with Dunnett’s multiple comparison’s test. (*p < 0.05, **p < 0.01, ***p < 0.001).

To examine the structural requirements for the ability of GML to modulate T cell function, the chain length, head group polarity, linkage, and lauric acid number or position were altered (Fig. 1). To this end, we acquired or synthesized compounds with chain lengths of 8 (8-CC), 10 (10-CC), 12 (GML), and 14 (14-CC) carbons. We also modified the glycerol head group of GML to be more hydrophobic by removing the hydroxyl groups from glycerol (lauric acid ethyl ester (LAEE) and isopropyl laurate (IPL)), or more charged by replacing the glycerol head group with a sugar (beta-d-glucopyranoside (OBDG), or carboxylic acid (GML Acid). The ester linkage was altered to an ether (ether) or an amide (GML Amide). Position of the laurate group was shifted to the second position in the 2-monolaurin, and dilaurin had a lauric acid in the 1 and 3 positions. Finally, we removed the glycerol head group leaving a 12-carbon chain carboxylic acid (lauric acid (LA)), or a 12-carbon chain primary amine (lauramide (LAM)). To examine these compounds, we used primary human activated peripheral blood T cells (APBTs). APBTs are T cells isolated from peripheral blood mononuclear cells that have been activated and expanded for treatment as described in the Methods. To test toxicity of these compounds, we treated APBTs with 10 ug/mL of each compound for 24 h (Fig. 2). The only compound that significantly impacted cell viability was the ether, which appeared to act as a detergent and dissolve cell membranes. Due to its toxicity, the ether analog was not used in further testing.

### Chain length, head polarity, and linkage moiety contribute to GML’s disruption of lipid membrane

Lipids in the plasma membrane are dynamic and exist on a spectrum of static ordered lipid domains to more fluid disordered lipid domains^17^. Clustering of the adaptor protein LAT, which is necessary for TCR-dependent T cell activation, is initiated by binding of a Grb2 dimer and further stabilized by fusion of ordered lipid raft domains^11,12,17–27^. Interestingly, previous studies observed that GML treatment increases the proportion of lipids in ordered and disordered regions in human T cells, suggesting that GML alters the dynamics of lipid domains^10^. To investigate the properties of GML that contribute to this effect, APBTs were treated with individual compounds or EtOH and then stained with Di-4-ANEPPDHQ, a dye with different fluorescent properties when it incorporates into ordered or disordered regions of cellular membranes. Changes in the fluorescence intensity of Di-4-ANEPPDHQ at different wavelengths were then measured by flow cytometry^10,28^.

**Figure 3:**
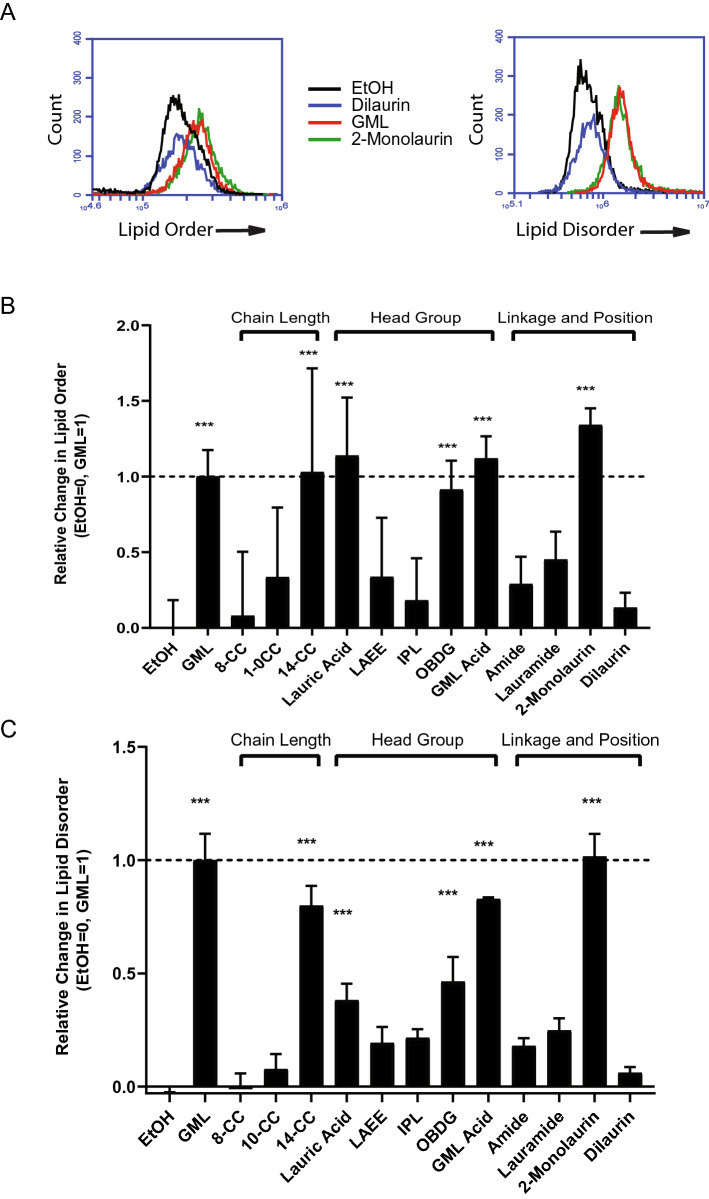
Changes in chain length, head group polarity, and linkage affect lipid membrane dynamics. APBT were treated with 10 ug/mL of designated compounds, stained with Di-4ANEPPDHQ**,** and analyzed on an Accuri C6 flow cytometer. Representative figures from data collection are presented with “Ordered” membrane and “disordered” membrane were analyzed using different fluorescent channels (**A**). Quantified relative change in “Lipid Order” (**B**) and “Lipid Disorder” (**C**) is compiled from 3 independent experiments with distinct donors and is presented as a fold change on a range of 0 to1, with EtOH set to 0 and GML set to 1. Statistical significance was determined using a Repeated Measures One-Way ANOVA. (*p < 0.05, **p < 0.01, ***p < 0.001).

Interestingly, the GML analog with 14 carbons in the acyl chain impacted order and disorder in T cell membranes similarly to GML, while analogs with an 8 or 10 carbon acyl chain had no effect on membrane dynamics (Fig. 3). GML analogs with polar head groups, GML Acid and OBDG, showed similar membrane effects as GML. Treatment with analogs containing non-polar headgroups, IPL and LAEE, and derivatives missing the glycerol head group, lauric acid and the lauramide, had no significant impact on the levels of membrane order and disorder in T cells (Fig. 3). Modification of the linkage of GML resulted in no changes in membrane lipid dynamics, with the amide having no impact on the levels of membrane order or disorder (Fig. 3). Finally, 2-monolaurin had the same effect on membrane dynamics as GML treatment, while adding a second laurate group resulted in elimination of effects on lipid membrane dynamics to levels similar to the EtOH control (Fig. 3). This indicates that a single laurate group with a 12 carbon or longer acyl chain and a charged head group attached by an ester linkage are required to alter lipid order and disorder in human T cells.

### GML requires specific chain length, head polarity, and linkage components to suppress early TCR-mediated protein cluster formation and calcium flux

**Figure 4:**
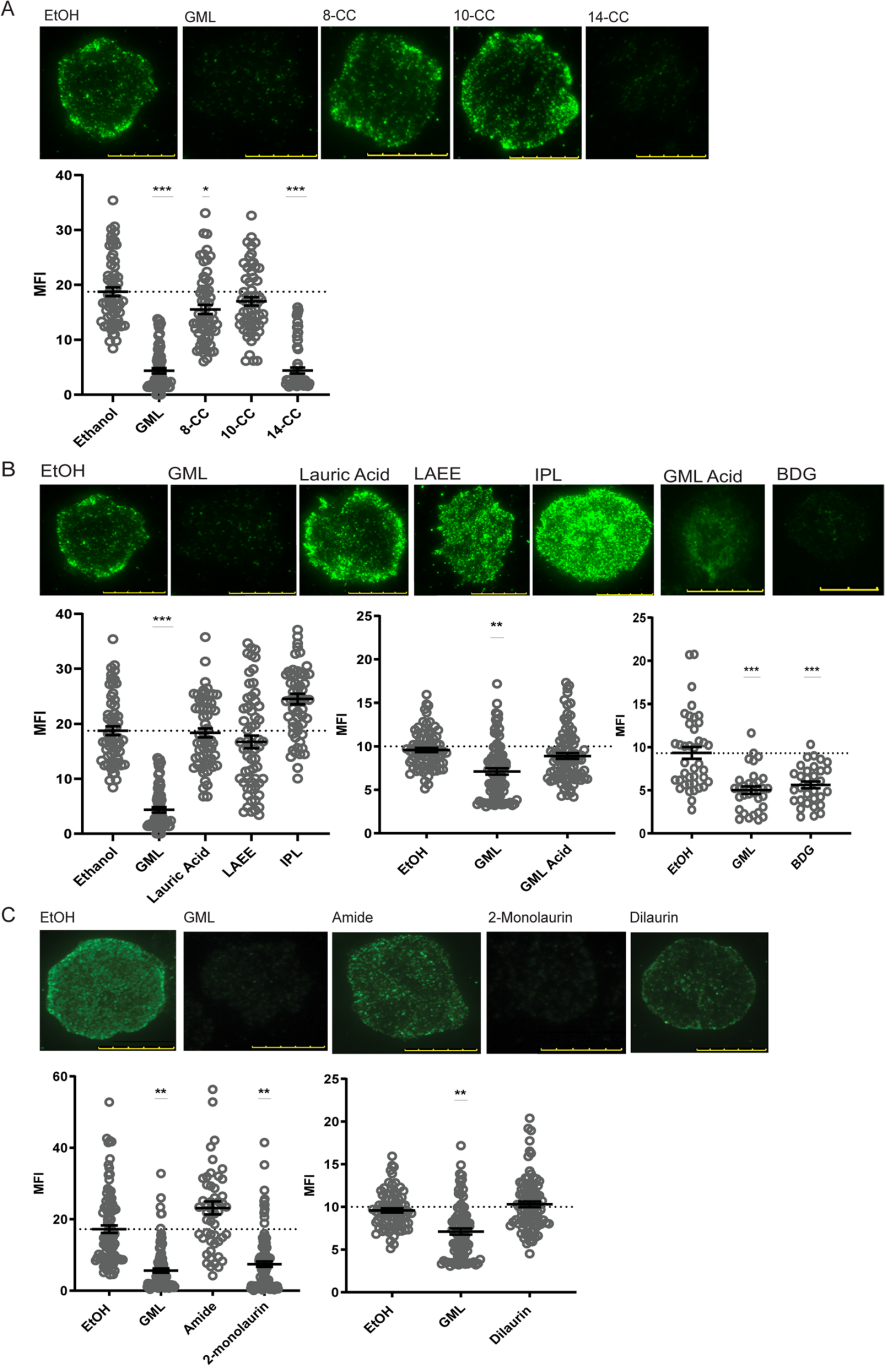
Chain length, head group polarity, and position impact the ability of GML to inhibit LAT clustering. APBTs were treated with 10ug/mL of designated compound and stimulated for 10 min using glass chamber slides coated with 10 ug/ml of anti-CD3. Cells were then fixed, permeabilized, and stained with antibodies specific for phosphorylated-LAT Y226. pLAT clustering in individual cells were imaged using TIRF. Representative individual cells are shown. Mean pixel intensities (MFI) of cells were quantified using ImageJ. Data compiled from at least 60 cells from 2 to 3 independent experiments are shown. The dotted line in each graph indicates MFI of EtOH, and data is plotted as mean with SEM. (**A**) Chain length (**B**) head polarity (**C**) linkage and Position (*p < 0.05, **p < 0.01, ***p < 0.001, scale bar = 10 µm).

**Figure 5:**
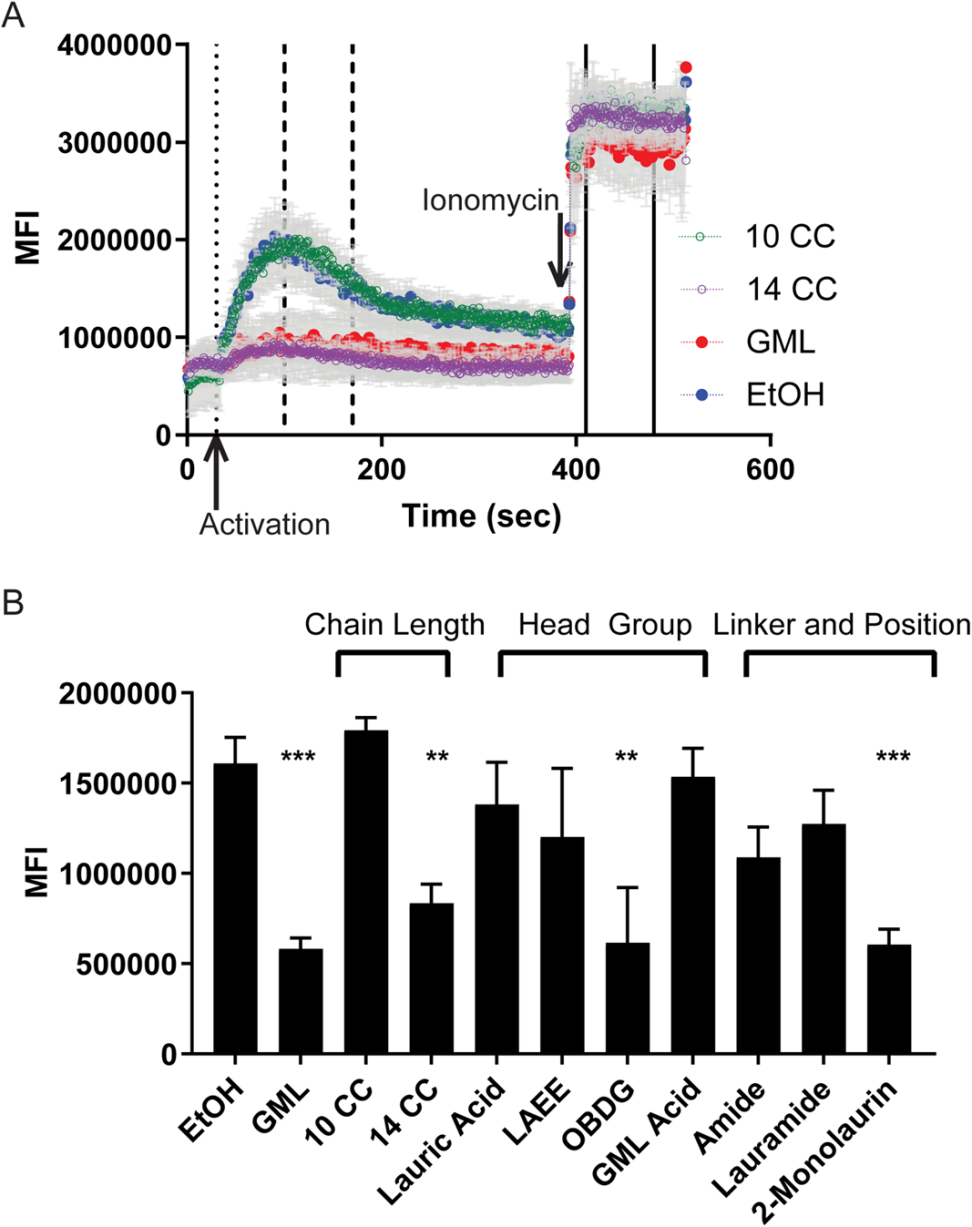
Chain length, head group polarity, and position impact the ability of GML to inhibit calcium signaling. Human APBTS were incubated with Fluo-4 AM cell permeable calcium dye and treated with designated compound. (**A**) Calcium flux was detected in real time using an Accuri C6 Flow cytometer. Baseline readings were taken for 30 s, cells were stimulated for 6 min, and finally were treated with Ionomycin to control for calcium dye levels. (**B**) Peak activation was averaged across 70 s (100–170 s post-stimulation) for each experiment. At least 3 independent experimental samples were compared to averaged control groups using a One-Way ANOVA with Dunnett’s multiple comparison’s test. (*p < 0.05, **p < 0.01, ***p < 0.001).

In addition to changing membrane lipid dynamics, GML treatment inhibits early TCR-induced pLAT clustering and calcium flux^10^. TIRF microscopy was used to measure the impact of GML analogs on pLAT clustering in APBTs (Fig. 4). To examine the effects of GML derivatives on TCR-mediated calcium influx, we measured the flow of calcium into stimulated APBTs in real time via flow cytometry using the calcium sensitive dye Fluo4AM. Figure 5a is representative of calcium influx data collection in real time with lines indicating segments for baseline influx, influx after activation, and influx after treatment with positive control. The bar graph shows averaged activation period for each compound.

Similar to the effects on membrane dynamics, treatment with derivatives with less than 12 carbons did not have a significant impact on TCR-mediated LAT clustering or calcium influx (Figs. 4a and 5b). Treatment with GML or 14-CC resulted in a significant decrease in pLAT clustering and calcium influx when compared to the EtOH treated control. Treatment with OBDG reduced pLAT clustering and calcium influx to a similar level as GML treatment. In contrast, treatment with LAEE and IPL, compounds with nonpolar head groups, had no impact on TCR-mediated pLAT clustering or calcium flux (Figs. 4b and 5b). Interestingly, lauric acid and GML Acid, both of which have substantial charge, had similar levels of pLAT clustering and calcium influx as the EtOH controls (Figs. 4b and 5b). Amide and Lauramide treatment had no significant effect on pLAT clustering and calcium influx compared to the EtOH controls (Figs. 4c and 5b). 2-monolaurin exhibited the same level of inhibition of both pLAT and calcium flux as GML treatment, while treatment with Dilaurin had no impact on pLAT clustering or calcium influx (Figs. 4c and 5b). From these data, we conclude that GML inhibition of pLAT clustering and calcium influx requires a 12-carbon or longer acyl chain linked to variable positions on a charged glycerol head group via an ester linkage.

### GML depends on carbon chain length, head polarity, and linkage to suppress cytokine production

**Figure 6:**
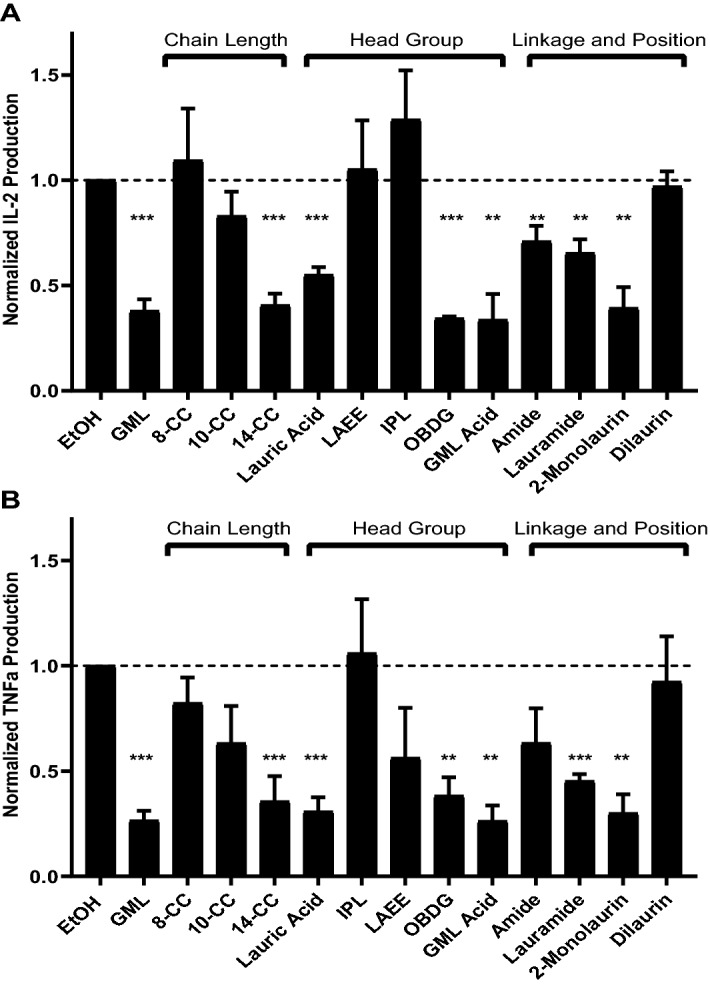
Chain length, head group polarity, and position affect the ability of GML to inhibit cytokine production. Rested human APBTs were treated overnight with 10 ug/mL of designated compound in serum free RPMI. Supernatants were collected and the release of (**A**) IL-2 and (**B**) TNF-α were assessed by ELISA. Values for each treatment were normalized to EtOH control to account for donor variability. Data for each compound was compiled from 3 to 5 donors. Dashed line indicates EtOH control cytokine production. One-sample T-test analysis was used to determine statistical significance. (*p < 0.05, **p < 0.01, ***p < 0.001).

Cytokine production is a key endpoint of T cell activation and the primary effector mechanism for CD4 T cells. TCR-induced cytokine production relies on lipid membrane interactions, pLAT clustering, and calcium influx. Previous studies have shown GML treatment inhibits TCR induced production of interferon-γ, TNF-α, IL-2, and IL-10 ^10^. To address the structural features of GML necessary to inhibit TCR induced cytokine production, APBTs were activated using plate bound anti-CD3 antibody and soluble anti-CD28 antibody and treated overnight with 1, 10, or 20 ug/mL of GML analogs. TNFα and IL-2 levels were measured from culture supernatants. Only the 10 ug/mL treatment data is presented (Fig. 6) because no compounds impacted cytokine production at 1ug/mL and all compounds that inhibited at 10 ug/mL also inhibited cytokine production at 20 ug/mL (data not shown).

Similar to previous findings, derivatives with an acyl chain of 12 carbons and 14-carbons significantly inhibited TCR-mediated production of IL-2 and TNFα. Derivatives with 8 and 10-carbon acyl chain had no significant impact on cytokine production compared to EtOH positive control groups (Fig. 6). Interestingly, compounds with polar, charged head groups, lauric acid, OBDG, and GML acid, all significantly reduced TCR-induced release of IL-2 and TNFα compared to control treatment (Fig. 6). However, treatment with analogs with non-polar head groups, IPL and LAEE, had no impact on cytokine production compared to EtOH controls (Fig. 6). Amide and lauramide, that have an amide linkage or primary amine, respectively, significantly suppressed IL-2 and TNF-α production compared to control treated cells (Fig. 6). Interestingly, treatment with 2-monolaurin inhibited IL-2 and TNF-α release to a similar level as GML, while dilaurin had no effect on cytokine production (Fig. 6). From these data, we conclude that a chain length of 12–14 carbons, a polar head group, and a single laurate group at any position are all key structural components that contribute to GML inhibition of TCR-induced cytokine production.

## Discussion

**Figure 7:**
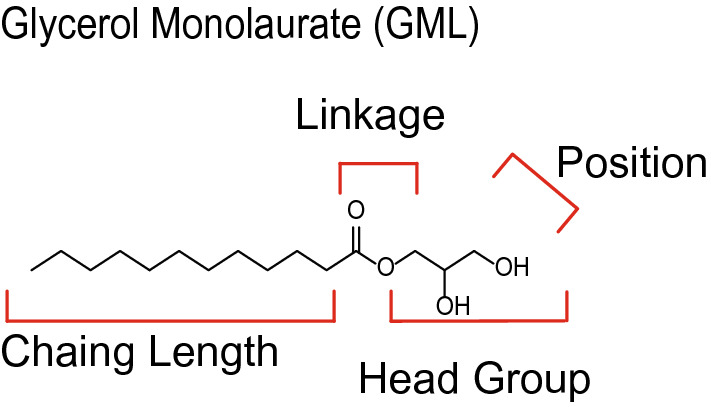
12-Carbon chain length, a polar head group, and oxygen linkage is required for the ability of GML to suppress T cell function. Carbon chain length of at least 12 carbons is required for GML inhibition of T cell activation. A polar head group and carbonyl linkage is necessary for disrupting membrane dynamics, protein clustering, and calcium signaling. Position of the laurate group does not affect GML activity, as 2-monolaurin is just as effective as GML at inhibiting T cell activity in all functional assays. However, more than a single laurate group causes the compound to become inactive and no longer suppress T cell function.

In this study, we addressed the components of GML that are necessary for its ability to inhibit T cell activation (Fig. 7). We found that at least 12-carbons in the acyl chain are needed to alter lipid membrane dynamics, protein clustering, calcium flux, and cytokine production. Analogs with an acyl chain that contains less than 12-carbons had no effect on these events. This suggests that a 12-carbon acyl chain is optimal for interfering with transmembrane signaling. We have previously shown that GML interferes with lipid membrane dynamics of human T cells^10,16^. A proposed mechanism for this disruption is GML’s interference with lipid rafts; thus, 12-carbons or more may be necessary for GML to interact with these structures. Interestingly, antimicrobial studies observed that GML compounds with shorter chain lengths can inhibit bacterial growth. Earlier studies suggest GML’s primary mechanism of bacterial inhibition is interference with signal transduction. Production of specific lipases and presence of the lipopolysaccharide outer layer of some bacterial cell membranes impact bacterial susceptibility to GML inhibition^29–31^. While the exact mechanism of how GML alters membrane dynamics is still an area of current investigation, these data suggest mechanisms of GML interactions with cells may differ between human T cells and bacteria^29,30^.

We observed that a polar head group is necessary to alter lipid membrane dynamics, inhibit protein clustering, and inhibit calcium flux, however there was some variability in this group of compounds tested. IPL and LAEE, compounds that are nonpolar, had no effect on TCR-induced T cell activation, while OBDG inhibited all investigated aspect of T cell activation in a manner similar to GML. However, certain compounds varied in how they impacted TCR-induced T cell activation. Lauric acid, amide, and lauramide did not affect lipid membrane dynamics, protein clustering, or calcium flux, but did inhibit TCR-dependent IL-2 and TNF-α production. Similarly, GML acid did not impact protein clustering or calcium influx but did affect lipid membrane dynamics and inhibited IL-2 and TNF-α production. Polarity and charge of head groups significantly impact how a molecule will interact and/or incorporate with lipids in the cell membrane. As mentioned previously, a proposed primary mechanism of GML inhibition is disrupting membrane dynamics by incorporating into the cell membrane and altering signal transduction^10,30^. Our data suggests a polar head group is necessary for this alignment, but it is possible that a highly charged head group, such as with lauric acid and GML acid, may interfere with a compound’s ability to incorporate into membrane structures.

The ester linkage is required for altering lipid membrane dynamics, inhibiting protein clustering, and inhibiting calcium flux. Interestingly, altering this ester linkage to an ether results in a cytotoxic compound. However, replacing the oxygen in this linkage with a nitrogen alters only part of the compound’s function. Lauramide and GML amide had no impact on membrane dynamics, protein clustering, or calcium signaling, however they did inhibit cytokine production. Ester, amide, and ether linkages all have unique stereochemistry. Thus, alterations to linkages of a compound will alter the 3-dimensional shape of the molecule and potentially change the mode of interaction with lipids in the cell membrane.

The laurate group can be in the 1st or 2nd carbon position and still alter lipid membrane dynamics, and inhibit protein clustering, calcium flux, and cytokine production. A single laurate group is required for any inhibition activity, more than one laurate group results in loss in activity. Dilaurin differs from most phospholipids found in the cell membrane because of the positioning of the acyl chains and head group. While most phospholipids have the acyl chains in the sn1 and sn2 positions, dilaurin has acyl chains in the sn1 and sn3 positions. It is possible these differences in structure prevent it from incorporating effectively into the cell membrane to inhibit T cell activation. Alternatively, if they can incorporate, the double acyl chains may provide dilaurin with a structure overly similar to endogenous membrane phospholipids and thus cannot significantly alter phospholipid interactions.

Lauric acid and amide raise an interesting question in response to previous mechanisms of action described for GML. These compounds inhibit cytokine production but do not alter the levels of order/disordered membrane lipid domains and do not inhibit protein clustering or calcium signaling. These data suggest GML may have an alternative mechanism to suppress cytokine production that is separate from its ability to alter lipid membrane dynamics and inhibit protein clustering. A consistent feature of GML in its interaction with human T cells and bacteria is the association with cellular membranes. It is likely that GML can diffuse completely through the plasma membrane, interacting and interfering with organelle membranes, thereby altering their function. Which specific organelles GML is incorporating in to and how it impacts overall cell function is an area of current research for our laboratory.

Furthermore, this project also addressed whether there are other compounds similar to GML that could work more effectively as an immune-modulating therapy. As discussed previously, none of the derivatives tested inhibited cellular function at lower doses than GML, however there were several derivatives that resulted in the same level of T cell inhibition with similar dosages as GML treatment. This is promising, as it provides evidence that GML has the potential to be altered and still be functional as an immune modulating molecule. Another factor to consider for pharmacological application is metabolic stability. Alterations to linkage and position, for instance, would inhibit the ability of endogenous lipases to metabolize GML. While a more metabolically stable molecule may come with its own unforeseen complications, it is an intriguing avenue to pursue.

In conclusion, we have identified several structural features of GML that are required for its ability to inhibit human T cell activation. We identified several analogs that suppressed function at the same concentration as GML treatments. These analogs had modifications that could contribute to a more metabolically stable compound for future investigation. Furthermore, several compounds tested inhibited the primary downstream function of cytokine production but did not change lipid membrane dynamics nor did they inhibit protein clustering or calcium signaling, suggesting mechanisms of action beyond altering lipid membrane dynamics. These findings expand our current understanding of GML’s mechanism of action and highlight that GML and similar compounds are a novel class of immunosuppressants.

## Materials and methods

### Compounds

GML analogs that varied in carbon chain length and head group were purchased from Caymen Chemical and ThermoFisher. Derivates further varying in head group, linkage, and position were synthesized by collaborators on this project in Dr. Kerns lab as detailed in supporting information. Compounds were reconstituted in ethanol at 100 ug/mL and stored at − 20 ºC.

### Primary human T cell isolation

PBMCs were isolated from LRS cones obtained from the DeGowin Blood Center at the University of Iowa Hospitals and Clinics. Donors are anonymous and have provided written informed consent to allow their cells that are normally discarded to be used in research studies. The recruitment protocol and written informed consent document were approved by the Institutional Review Board for the University of Iowa. All samples were provided to investigators de-identified. Therefore, further IRB approval for the use of the cells by the investigators was not needed based on Federal Regulation 46.101.B4. Hence, all experiments were performed in accordance with approved guidelines. PBMCs were isolated using gradient separation with LymphoPrep (Stem Cell Technologies). T cells were incubated with bead bound anti-CD3 and anti-CD28 antibodies (Invitrogen) for 5 days of activation and expansion. Cells were then resuspended in fresh media overnight without stimulatory agents. This cell population contains a mix of CD4 and CD8 T cells and is termed activated peripheral blood T cells (APBTs).

### Cytotoxicity assays

APBTs were incubated overnight with 10ug/mL of designated compound in serum free RPMI media (ThermoFisher). Cell viability was determined using Trypan Blue exclusion assay. APBTs were mixed with 50 mM Trypan Blue solution. Trypan Blue positive and Trypan Blue negative cells were counted using an Olympus CKX41 microscope with a 10 × objective. Percent viability was calculated using the formula (Trypan Blue Neg. Cells)/(Trypan Blue Pos. + Trypan Blue Neg.). Data was compiled from 3 independent experiments with distinct donors. Statistical significance was determined using a Repeated Measures One-Way ANOVA with Dunnett’s multiple comparison’s test.

### Membrane order versus disorder measurement

APBTs were washed and resuspended in serum free RPMI without phenol red. Cells were treated with designated lipid or EtOH loading control for 10 min at 37 °C. Cells were then stained with 1 uM Di-4ANEPPDHQ and mean fluorescence was analyzed using an Accuri C6 Flow Cytometer. “Ordered” membrane was read in FL1 channel and “disordered” membrane was read in the FL3 channel. Data is presented as a fold change on a range of 0 to1, with the fluorescent value for EtOH set to 0 and the fluorescent value for GML set to 1. Data was compiled from 3 individual experiments with distinct donors. Statistical significance was determined using a Repeated Measures One-Way ANOVA.

### Total internal fluorescent microscopy (TIRF)

APBTs were treated with 10 ug/mL of designated lipid, or EtOH, and stimulated with plate-bound anti-CD3 antibody in serum-free RPMI without phenol red in Coverglass 4-well Chamber Slides. After 10 min of stimulation, cells were fixed with 4% paraformaldehyde for 30 min. After washing and blocking, the samples were incubated overnight at 4 °C with mouse anti-LAT pY226 antibody (J96-1238.58.93, BD Biosciences). Alexa-Fluor 488 goat anti-mouse IgG1 antibody (polyclonal, Invitrogen) was incubated with the fixed cells for 2 h at 37 °C. After washing, cells were immediately imaged using a Leica AM TIRF microscope with a 100 × magnification oil immersion objective lens at the University of Iowa Central Microscopy Research Facility. LAT clustering was quantified analyzing the mean fluorescent intensity (MFI) along a single line drawn across the widest point of each cell using ImageJ software. MFI for each compound was compiled from at least 60 cells from 2 to 3 independent experiments using different donors. Statistical significance was determined using a one-way ANOVA with Dunnett’s multiple comparisons test.

### Calcium flux assays

APBTs were incubated with Fluo-4 AM cell permeant calcium dye (LifeTechnologies) and Probenocid (LifeTechnologies). Cells were then treated with 10 ug/mL of designated lipid or EtOH loading control, anti-CD3 and anti-CD4 antibodies, and incubated on ice for 15 min. Cells were warmed for 5 min at 37 °C before being analyzed on an Accuri C6 Flow cytometry. Baseline cytoplasmic calcium levels were taken for 30 s. Calcium influx in response to T cell stimulation was analyzed in real time by stimulating cells with addition of IgG crosslinking antibody for 6 min. Ionomycin was then added as a control for total Fluo-4 AM levels. Mean fluorescent values were averaged across at least 3 independent experiments from 3 different donors. Baseline was averaged from the first 30 s, peak activation was averaged from 100 to 170 s (70–140 s post-stimulation), and Ionomycin control was averaged from 410 to 480 s (380–450 post-stimulation). Experimental samples were compared to averaged control groups using a One-Way ANOVA with Dunnet’s multiple comparisons post-hoc test.

### Cytokine production assays

APBTs were treated with 1ug/mL, 10 ug/mL, or 20 ug/mL of designated lipid or EtOH loading control and stimulated overnight in serum-free RPMI with plate-bound anti-CD3 antibody (OKT3, BioLegend) and soluble anti-CD28 antibody (CD28.2, BioLegend). Supernatant was collected, and cytokine production was measured via standard Enzyme Linked Immuno Assay (ELISA). IL-2 was measured using an anti-IL2 antibody (MQ1-17H12, eBioscience) and biotin conjugated IL-2 antibody (polyclonal, eBioscience). TNF-α was measured using an anti-TNF-α antibody (MAb1, BioLegend) and biotin conjugate TNF-α antibody (MAb11, BioLegend). Cytokine production was normalized to EtOH positive control for each experiment to control for donor variability. Statistical significance was measured using a one-sample T-test comparing averaged experimental fold change to 1. Data was compiled from 3 to 6 independent experiments with different donors.

## Supplementary Information

Supplementary Information 1.
