# Supplementary material for: Suppression of human T cell activation by derivatives of glycerol monolaurate
Source: Sci Rep. 2021 Apr 26;11:8943. doi: 10.1038/s41598-021-88584-y (PMC8076190; doi:10.1038/s41598-021-88584-y)
Supplement: Supplementary file 1 — Supplementary Information 1. [file 41598_2021_88584_MOESM1_ESM.pdf]

Supplemental Information:

Suppression of Human T Cell Activation by Derivatives of Glycerol Monolaurate

Micaela G. Fosdick[1], Pratik Rajesh Chheda[3], Phuong M. Tran[2], Alex Wolff[2], Ronal Peralta[2], Michael Y. Zhang[2], Robert Kerns[3] and Jon C.D. Houtman[1,2,#]

[1]Biomedical Sciences Graduate Program, Subprogram in Molecular Medicine, Carver College of Medicine, University of Iowa

[2]Department of Microbiology and Immunology, Carver College of Medicine, University of Iowa

[3]Department of Pharmaceutical Sciences and Experimental Therapeutics, College of Pharmacy, University of Iowa

# Corresponding author: Jon Houtman
2110 MERF
Iowa City IA 52242
Jon-houtman@uiowa.edu

Supplemental Materials and Method:

General Information: All commercially available reagents and solvents were used directly without further purification unless otherwise noted. The following compounds were purchased from respective vendors: 1-Oxododecyl beta-d-glucopyranoside (OBDG) (Combi-Blocks, CAS #64395-92-2), Lauramide (Ark Pharm, CAS #1120-16-7), Dilaurin (Combi-Blocks, CAS #539-93-5) Lauric Acid (Cayman Chemical Company, CAS #143-07-7), Lauric Acid Ethyl Ester (Cayman Chemical Company, CAS #106-33-2), Isopropyl laurate (Sigma-aldrich, CAS#10233-13-3), 1-Octanoyl-*rac*-glycerol (Sigma-aldrich CAS #502-54-5), 1-Decanoyl-*rac*-glycerol (Sigma-aldrich, CAS# 2277-23-8). Reactions were monitored either by thin-layer chromatography (carried out on silica plates, silica gel 60 F_{254}, Merck) and visualized under UV light or using appropriate stains. Flash chromatography was performed using Silica Gel 60 purchased from EMD. ^{1}H NMR and ^{13}C NMR spectra were recorded in $CDCl_3$ or MeOD on a Bruker Avance spectrometer operating at 300 MHz at ambient temperature. All peaks are reported in ppm on a scale downfield from TMS and using the residual solvent peak in $CDCl_3$ (H δ = 7.26 ppm) or MeOD (H δ = 3.31 ppm) or TMS (δ = 0.0) as an internal standard. Data for ^{1}H NMR are reported as follows: chemical shift (ppm, scale), multiplicity (s =singlet, d = doublet, t = triplet, q = quartet, m = multiplet and/or multiplet resonances, dd = double of doublets, dt = double of triplets, bs = broad singlet), coupling constant (Hz), and integration. All low-resolution mass spectra (HRMS) were measured on Waters Premier mass spectrometer using electrospray ionization (ESI) time-of-flight (TOF).

Scheme:

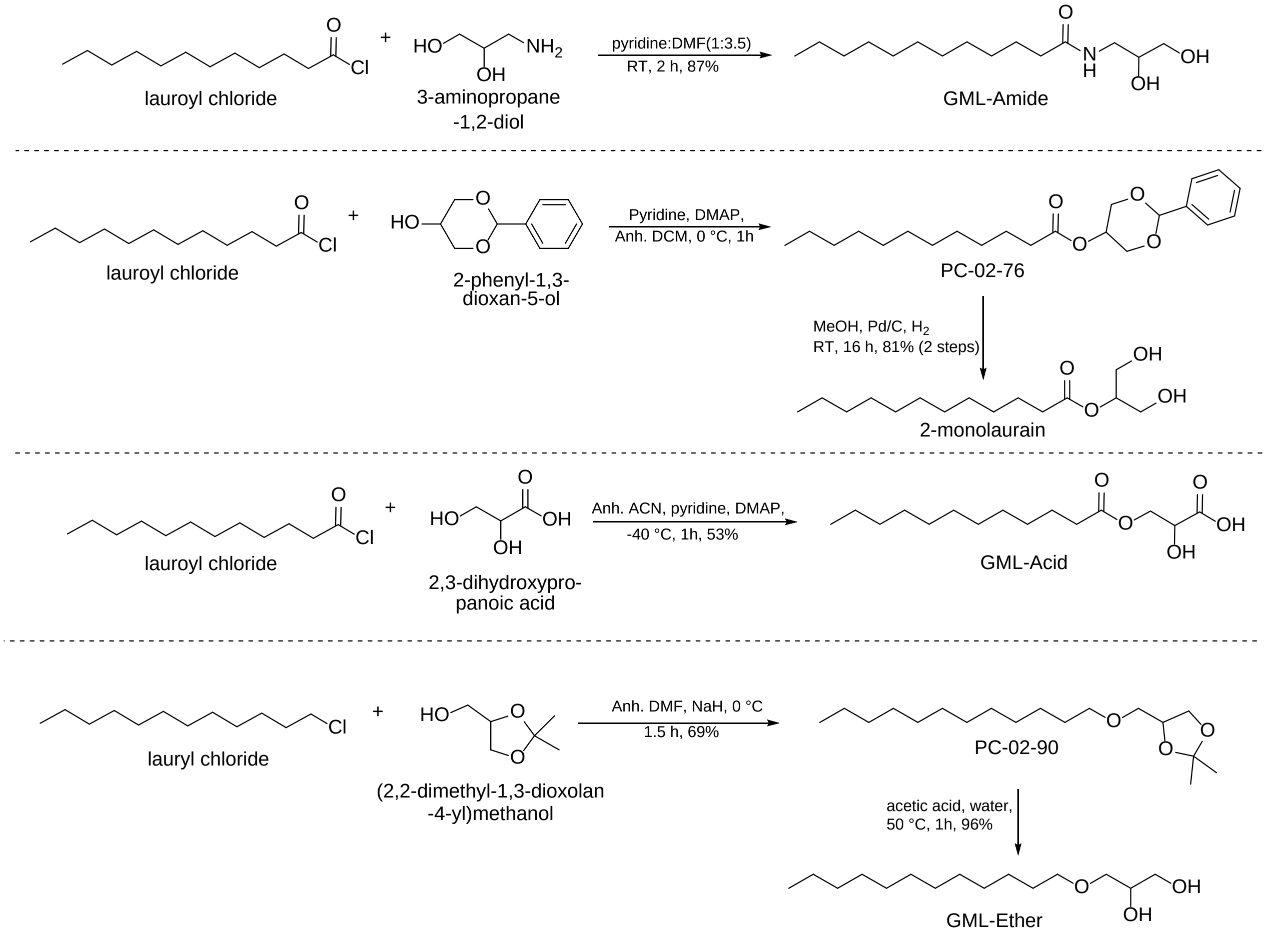

Synthetic procedure:

N-(2,3-dihydroxypropyl)dodecanamide (**PC-02-71/GML-amide**): To a stirring solution containing 3-aminopropane-1,2-diol (204 mg, 2.24 mmol, 1.3 equiv) in a solvent mixture of pyridine:DMF (1:3.5) was added lauroyl chloride (356 µl, 1.72 mmol, 1.0 equiv.) dissolved in DMF (2 ml) and the reaction was allowed to stir at RT for 2 hours. After 2 hours, the solvent was removed using rotavap to yield a white solid. The white solid was suspended in hexanes and sonicated for 5 minutes followed by filtering the solid. The solid was then suspended in water and sonicated again for 5 minutes. The resulting solid was filtered and dried under vacuum to provide compound **PC-02-71** as a white solid (368 mg, 87%). 1H NMR (300 MHz, MeOD) δ 3.75 – 3.64 (m, 1H), 3.49 (dd, J = 5.4, 1.5 Hz, 2H), 3.38 (d, J = 5.0 Hz, 1H), 3.20 (dd, J = 13.8, 6.7 Hz, 1H), 2.33 – 2.10 (m, 2H), 1.70 –

1.52 (m, 2H), 1.28 (d, J = 19.5 Hz, 16H), 0.92 (t, J = 6.7 Hz, 3H). LRMS (ESI), *m*/*z* calcd for $C_{15}H_{32}NO_3$ $[M + H]^+$ 274.24, found 274.24.

1,3-dihydroxypropan-2-yl dodecanoate (**PC-02-77/2-monolaurain**): To solution of 2-phenyl-1,3-dioxan-5-ol (100 mg, 0.55 mmol, 1.0 equiv), pyridine (54 µl, 0.66 mmol, 1.2 eq.) and 4-dimethylaminopyridine (10 mg, 10 mol%) in anhydrous dichloromethane (5 ml) was allowed to stir at 0 ℃ for 10 minutes. To this was added lauroyl chloride (155 µl, 0.66 mmol, 1.2 equiv.) and the resulting solution was allowed to stir at RT for 1 hour. After 1 hour, the reaction was diluted with dichloromethane (20 ml) and washed with 20 ml of 4N HCl followed by 20 ml of saturated sodium bicarbonate solution. The organic fractions were collected, concentrated and purified by column chromatography using 0-20% hexanes:ethyl acetate as eluent to give **PC-02-76 (2-phenyl-1,3-dioxan-5-yl dodecanoate)** as a clear oil which was used for next step. To a stirring solution of **PC-02-76** (180 mg) in methanol (4 ml) was added palladium on carbon (18 mg, 10 mol%) and the RBF was sealed and back filled three times with hydrogen gas and then the reaction was allowed to stir for 16 hours under positive pressure of hydrogen. After 16 hours, the reaction was diluted with methanol and the palladium on carbon was removed by filtration. The filtrate was concentrated to furnish **PC-02-77** as a white solid (124 mg, 81% over 2 steps). ^1H NMR (300 MHz, $CDCl_3$) δ 4.92 (s, 1H), 3.82 (s, 3H), 3.26 (s, 2H), 2.58 – 2.10 (m, 2H), 1.63 (s, 2H), 1.26 (s, 16H), 0.88 (t, *J* = 6.7 Hz, 3H). LRMS (ESI), *m*/*z* calcd for $C_{15}H_{30}O_4Na$ $[M + Na]^+$ 297.20, found 297.20.

3-(dodecanoyloxy)-2-hydroxypropanoic acid (**PC-02-91/GML-acid**): To a stirring suspension of 2,3-dihydroxypropanoic acid (100 mg, 0.94 mmol, 1.0 equiv.) in anhydrous acetonitrile (2 ml) was added pyridine (46 µl, 0.56 mmol, 0.6 equiv.) and 4-dimethylaminopyridine (10 mg, 10 mol%) and the resulting suspension was allowed to stir at -40 ℃ for 20 minutes. To this was added dropwise a solution of lauroyl chloride (130 ml, 0.56 mmol, 0.6 equiv.) in acetonitrile (500 µl) and the resulting suspension was allowed to slowly warm to 0 ℃. Once at 0 ℃, the reaction was quenched water (10 ml) and extracted with ethyl acetate which was washed thrice with water. The organic fractions were concentrated to provide **PC-02-91** as a white solid (145 mg, 53%). ^1H NMR

(300 MHz, $CDCl_3$) δ 6.17 (s, 2H), 5.21 (dd, *J* = 4.2, 3.4 Hz, 1H), 4.07 (ddd, *J* = 19.3, 12.3, 6.6 Hz, 2H), 2.46 (td, *J* = 7.5, 2.5 Hz, 2H), 1.67 (dd, *J* = 14.7, 7.3 Hz, 2H), 1.44 – 1.14 (m, 16H), 0.90 (t, *J* = 6.7 Hz, 3H). LRMS (ESI), *m/z* calcd for $C_{15}H_{28}O_5Na$ $[M + Na]^+$ 311.18, found 311.18.

3-(dodecyloxy)propane-1,2-diol (**PC-02-95/GML-ether**): To a stirring solution of (2,2-dimethyl-1,3-dioxolan-4-yl)methanol (155 mg, 1.17 mmol, 1.2 equiv.) in anhydrous DMF (2 ml) at 0 ℃ was added sodium hydride (35 mg, 1.47 mmol, 1.5 equiv.) and the resulting suspension was allowed to stir at 0 ℃ for 20 minutes. To this was added laurylchloride (230 µl, 0.97 mmol, 1 equiv.) and the reaction was allowed to stir for 90 minutes. Once complete, the reaction was quenched with water (10 ml) and extracted with ethyl acetate. The organic fraction was concentrated and purified by column chromatography using 0-20% hexanes:ethylacetate as eluent to give **PC-02-90 (4-((dodecyloxy)methyl)-2,2-dimethyl-1,3-dioxolane)** (203 mg, 69%) as a clear oil and was used for the next step. To **PC-02-90** (203 mg) was added acetic acid (4 ml) and water (1 ml) and the resulting solution was allowed to stir at 50 ℃ for 1 hour. Once complete, the reaction was neutralized with saturated sodium bicarbonate and then extracted with ethyl acetate. The organic fractions were concentrated to furnish **PC-02-95** (248 mg, 96 %) as a white solid. ^1H NMR (300 MHz, $CDCl_3$) δ 3.87 (d, *J* = 4.4 Hz, 1H), 3.77 – 3.37 (m, 6H), 2.75 (s, 1H), 2.36 (s, 1H), 1.58 (dd, *J* = 13.6, 6.7 Hz, 2H), 1.39 – 1.17 (m, 18H), 0.89 (t, *J* = 6.7 Hz, 3H). LRMS (ESI), *m/z* calcd for $C_{15}H_{32}O_3Na$ $[M + Na]^+$ 283.25, found 283.25.
